# An Exploration of Patient Experiences Concerning Coercive Measures: A Qualitative Study in Closed Psychiatric Settings in Iran

**DOI:** 10.1111/hex.70514

**Published:** 2025-11-27

**Authors:** Hamid Asayesh, Mohammad Parvaresh‐Masoud, Vahideh Nayeri, Ali Khaji, Ahmad Mashkoori

**Affiliations:** ^1^ Department of Philosophy and Ethics of Health, School of Health and Religion Qom University of Medical Sciences Qom Iran; ^2^ Department of Medical Emergencies, School of Allied Medical Sciences Qom University of Medical Sciences Qom Iran; ^3^ Department of Psychiatry, School of Medicine Qom University of Medical Sciences Qom Iran; ^4^ Iranian Research Center on Aging University of Social Welfare and Rehabilitation Sciences Tehran Iran; ^5^ Spiritual Health Research Center, School of Health and Religion Qom University of Medical Sciences Qom Iran

**Keywords:** closed psychiatric settings, coercive measures, patient experiences, psychiatric inpatients

## Abstract

**Introduction:**

Coercive measures in psychiatry are sometimes necessary to prevent harm, but raise significant ethical and clinical concerns and can negatively impact patients. Despite extensive research on coercion in Western settings, studies from the Middle East, particularly Iran, remain scarce, leaving gaps in understanding the cultural and structural factors influencing coercion. This study examines the experiences of patients subjected to formal and informal measures in locked psychiatric wards in Iran.

**Methods:**

A qualitative content analysis was conducted involving 13 patients from locked psychiatric wards. Participants were selected through purposive sampling to ensure a diverse range of experiences with coercive measures. Data were collected using semi‐structured interviews, allowing for a comprehensive understanding of patients' experiences, feelings and perceptions of coercion.

**Results:**

The data analysis revealed three primary themes, each of which has sub‐themes that enhance understanding of the participants' experiences. (1) Experiences of coercion: Patients reported experiencing trauma from physical and mechanical restraint, as well as the coercive atmosphere of the wards. Patients reported alternatives to coercive measures, emphasising therapeutic relationships, addressing irritability causes and flexible ward routines as key preventive strategies. (2) Roots of coercion: Poor staff communication, patient disturbing behaviours and restrictive environments were key triggers prompting staff to use coercive measures. (3) Consequences of coercive measures on patients: Psychological distress (e.g., fear, anger and suicidal ideation), distrust in care and violations of dignity were reported as negative consequences of coercive measures.

**Conclusion:**

This study highlights the intricate nature of patient experiences with coercive measures in psychiatric settings. The findings underscore the need for improved provider–patient communication and the implementation of alternative approaches that prioritise patient dignity and autonomy. Addressing these issues is crucial for enhancing the quality of psychiatric care and minimising the reliance on coercive practices.

**Patient or Public Contribution:**

This study was primarily informed by the perspectives and experiences of psychiatric patients with regard to coercive measures. Their direct participation provided essential data for analysis and findings. Patients participated in the study through in‐depth interviews, which ensured that the research captured authentic narratives about the impacts of coercion, preventive alternatives and systemic challenges. These narratives formed the basis of the findings and guided the ethical and clinical implications discussed in the manuscript. The study design and conclusions are deeply rooted in patients' experiences and prioritise their voices when identifying areas for improvement in psychiatric care practices.

## 1 Introduction

The use of coercive measures in psychiatric settings remains a complex and controversial issue, raising serious ethical, therapeutic and practical concerns [1]. Coercive measures include both formal interventions, such as physical restraint, seclusion, forced medication and involuntary hospitalisation and informal pressures, such as threats, persuasion, manipulation or other subtle forms of coercion that may occur within therapeutic interactions [2, 3]. While these measures are sometimes implemented to ensure safety or manage crises, growing evidence shows that they can harm patients by reducing trust, undermining satisfaction with care and impairing therapeutic relationships [4, 5]. Coercion can also increase psychological distress, hinder recovery and exacerbate existing mental health conditions [6, 7]. Moreover, coercive practices have been associated with re‐traumatisation, feelings of shame and stigmatisation, post‐traumatic stress symptoms, and avoidance of future mental health care [8, 9]. Physical consequences have also been reported, particularly in relation to formal coercive measures such as restraint and seclusion, including bruises, muscle injuries, circulation problems and, in rare cases, serious medical complications or fatalities [10].

Patients' experiences of coercion are complex and multidimensional. Many report feelings of fear, humiliation and loss of autonomy, alongside occasional perceptions of safety or protection [11, 12]. Informal forms of coercion, such as subtle pressures or manipulative persuasion, often interact with formal interventions to shape patients' overall perceptions of their treatment. Exploring patients' subjective experiences provides critical insight into how coercion affects their sense of autonomy, dignity and trust—dimensions that cannot be fully captured through quantitative measures or staff‐reported data. A comprehensive understanding of this experiential spectrum is vital for developing patient‐centred interventions and therapeutic contexts that preserve safety while minimising potential harm [2, 6].

Most research on coercive measures originates from Western countries, including Australia, North America and Europe [13]. In contrast, knowledge from Asian and African contexts remains limited, and even though countries such as Japan and South Korea have begun to investigate these practices, research in the Middle East is particularly scarce [2, 14].

Cultural, legal and institutional factors strongly influence the use and perception of coercion. Systems that emphasise patient autonomy and rights generally impose stricter limits on coercive practices and often adopt alternatives such as open‐door ward policies, which are associated with lower rates of restraint and seclusion [15, 16]. In addition, a growing body of evidence supports the implementation of evidence‐based alternatives to both formal and informal coercive practices in psychiatric settings. Specific interventions such as the Safewards model, de‐escalation training programmes and sensory modulation rooms have been shown to reduce aggression, enhance communication and decrease the need for coercive measures [17]. Approaches grounded in trauma‐informed and recovery‐oriented care, as well as shared decision‐making, further promote mutual trust and respect between patients and staff. Collectively, these strategies contribute to creating safer, more collaborative and less coercive treatment environments [18]. Against this backdrop, the present study investigates the experiences of psychiatric inpatients subjected to both formal and informal coercive measures in locked wards in Iran. By exploring how patients perceive and interpret coercion, this study aims to inform more ethical, effective and compassionate psychiatric care practices.

## 2 Methods

Given the exploratory nature of the research and the aim to understand patients' experiences of coercion, a qualitative design was chosen as the most suitable approach. Conventional content analysis, as described by Graneheim and Lundman, was selected for its ability to capture both manifest and latent meanings in participants' narratives [19].

### 2.1 Study Context

This study was conducted in three locked adult psychiatric wards of a teaching hospital affiliated with Qom University of Medical Sciences in Iran. In Iran, the majority of inpatient psychiatric care is delivered in locked wards, whereas community‐based and open psychiatric services remain limited. A comprehensive national mental health law has not yet been enacted; however, certain civil regulations permit involuntary admission to psychiatric units under emergency circumstances.

Although empirical research on the topic is limited, available evidence indicates a relatively high prevalence of both physical and chemical restraints, with chemical restraints being more frequently employed. Reliable data on seclusion practices are scarce; nonetheless, within the wards included in the present study, seclusion was utilised only minimally. In clinical practice, orders for chemical restraint are typically issued and documented by psychiatrists to manage acute behavioural emergencies. Decisions concerning the application of physical or mechanical restraints are often made by nursing staff in response to critical situations and are generally recorded retrospectively.

At the national level, there are currently no standardised clinical guidelines governing the use of coercive interventions in Iran. Instead, locally developed protocols are occasionally implemented, although these often lack formal legal or regulatory authority. The clinical culture within such settings is characterised by a pronounced medical hierarchy, wherein psychiatrists' decisions carry substantial authority, and nurses are primarily responsible for executing prescribed treatment plans. Persistent nursing staff shortages and excessive workloads contribute to occupational stress and limit opportunities for reflective, patient‐centred practice. Collectively, these contextual factors shape the everyday dynamics of psychiatric care and influence how coercive measures are perceived, enacted and experienced by both patients and healthcare providers.

### 2.2 Participants

Participant recruitment and data collection were conducted between October 2024 and February 2025 in the psychiatric inpatient wards of Khayerin Salamat Hospital, affiliated with Qom University of Medical Sciences, Iran. Participants were selected through purposive sampling to capture a wide range of experiences regarding coercion, including variations in gender, diagnosis, type of hospitalisation (voluntary or involuntary) and type of coercive measures (e.g., physical or mechanical restraint, chemical restraint and seclusion). Eligible patients were identified by the ward psychiatrist once clinical stability had been achieved and were subsequently approached by an independent researcher, who provided information about the study and invited them to participate. All interviews were conducted face‐to‐face in a private room within the hospital, typically within the last days of hospitalisation. Inclusion criteria were: (1) age > 18 years; (2) having experienced at least one formal coercive measure during the current or recent hospitalisation; and (3) clinical stability at the time of interview. Clinical stability, cognitive functioning and insight into illness were evaluated by the treating psychiatrist through clinical assessment and mental status examination, in line with DSM‐5 clinical descriptions and diagnostic framework. These evaluations ensured that participants were able to engage meaningfully in a semi‐structured qualitative interview. All assessments were reviewed and confirmed by an independent psychiatrist before each interview. At the beginning of every session, the interviewer also evaluated participants' ability to communicate coherently and reflect on their experiences; individuals who did not meet these criteria were excluded. The demographic and clinical characteristics of the patients who participated in the study are shown in Table 1.

**Table 1:** Demographic and clinical characteristics of patients participating in the study.

Patients No.	Gender	Age (year)	Marital status	Education	Diagnosis	Substance dependency	Number hospitalisations	Current type of hospitalisation	Physical/mechanical restraint	Seclusion	Forced medication
1	Female	37	Single	Bachelor	Bipolar I	No	4	Involuntary	Yes	No	Yes
2	Male	48	Single	Diploma	Schizophrenia	No	1	Involuntary	No	No	Yes
3	Male	46	Married	High school	Bipolar I	Yes	3	Voluntary	Yes	Yes	Yes
4	Male	33	Single	Diploma	Bipolar I	No	2	Involuntary	Yes	No	Yes
5	Female	25	Married	Bachelor	Schizoaffective	No	1	Involuntary	Yes	No	Yes
6	Male	27	Single	Bachelor	Substance‐ induced psychosis	Yes	1	Involuntary	Yes	No	Yes
7	Male	35	Single	Diploma	Major depression	Yes	1	Voluntary	Yes	No	No
8	Male	30	Single	Diploma	Substance‐ induced psychosis	Yes	3	Involuntary	Yes	Yes	Yes
9	Male	34	Single	Diploma	Bipolar I	No	3	Involuntary	No	No	Yes
10	Male	24	Single	University student	Schizophrenia	No	2	Involuntary	Yes	No	Yes
11	Female	36	Married	Bachelor	Schizoaffective	No	1	Involuntary	Yes	No	Yes
12	Female	27	Married	Diploma	Bipolar I	No	5	Involuntary	Yes	Yes	Yes
13	Female	22	Single	Diploma	Schizophrenia	No	1	Involuntary	No	No	Yes

For the purpose of this study, ‘coercive measures’ were defined in two categories: Formal coercive measures, referring to legally sanctioned and institutionally regulated interventions such as involuntary admission, physical restraint, forced or intramuscular medication without consent, and seclusion. Informal coercive measures, referring to interpersonal or situational pressures exerted on patients without legal mandate, including persuasion, threats of negative consequences, withholding information or emotional pressure that may limit a patient's sense of autonomy or choice. Although the interviews primarily focused on experiences of formal coercion, patients were also encouraged to describe any perceived informal pressures or influences related to their hospitalisation or treatment.

### 2.3 Data Collection

Semi‐structured in‐depth interviews were conducted with each participant to capture rich, subjective accounts of their experiences with coercion in psychiatric wards. The interview guide, developed based on a literature review and expert input, was refined using feedback from two pilot interviews to ensure clarity and relevance. It comprised primarily open‐ended questions to elicit personal narratives, with probing questions employed as needed to explore specific experiences in greater depth. The interviews focused on formal coercive measures (e.g., involuntary admission, restraint, forced medication and seclusion) and also encouraged participants to share experiences of informal coercion, including interpersonal pressures, persuasion or perceived threats. This ensured a comprehensive capture of coercive experiences. Table 2 summarises the interview questions. All interviews were conducted face‐to‐face in private hospital rooms, typically during the final days of hospitalisation. Field notes were taken during and immediately after each interview to record contextual details, nonverbal expressions and the emotional tone of participants' narratives, which were used to supplement the interview data and support interpretation during analysis.

**Table 2:** Semi‐structured interview guide on patients' experiences of coercive measures in psychiatric wards.

No.	Interview question	Objective/Focus	Note on scope
1	Can you describe your overall experience with coercive measures during your time in the psychiatric ward?	Explore general perceptions and feelings about coercive interventions.	Participants could discuss both formal and informal coercion.
2	What specific coercive measures did you experience (e.g., seclusion, restraint and forced medication)?	Identify types of formal coercion encountered.	Also open to sharing experiences of informal pressures or interpersonal coercion.
3	How did you feel at the moment these measures were applied?	Understand immediate emotional responses.	Includes reactions to any perceived coercive actions.
4	What emotions did you experience during and after the use of coercive measures?	Explore short‐ and longer‐term emotional impact.	Both formal and informal coercion considered.
5	How do you think these experiences have affected your mental health and self‐perception?	Examine perceived psychological consequences.	Refers to all coercive experiences.
6	Do you believe that the coercive measures used were necessary for your treatment? Why or why not?	Capture the patient's perspective on necessity and justification.	Applies to any type of coercive experience.
7	How would you describe your interactions with staff members during the application of coercive measures?	Understand the relational context of coercion.	Includes both formal and informal interactions.
8	Did you feel listened to or respected by the staff when coercive measures were being applied? Why or why not?	Assess perceived respect, agency and dignity.	Covers experiences of informal pressures as well.
9	In your opinion, what could have been done differently to avoid the use of coercive measures?	Explore suggestions for prevention or improvement.	Applicable to formal and informal coercion.

### 2.4 Data Analysis

The data obtained from the interviews were analysed using the Granheim and Lundman method of content analysis [19]. This method entails the following steps: (1) the transcribed interviews were prepared. After each interview, the recorded audio of each session was meticulously transcribed verbatim. (2) Initial Coding: The transcripts were meticulously reviewed multiple times to identify significant statements related to coercion. These statements were then coded into categories reflecting common themes. (3) Theme Development: The codes were grouped into broader themes that encapsulated the essence of participants' experiences. This qualitative content analysis facilitated comprehension of the nuances of coercion as perceived by patients.

The analysis was conducted by two researchers (a psychiatric nurse and a psychiatrist) who independently coded all interview transcripts. A third researcher, a senior nursing scholar with extensive experience in qualitative research, reviewed the coding framework and thematic structure to ensure analytical rigour and consistency. Discrepancies were discussed among the team until consensus was achieved.

### 2.5 Trustworthiness

To ensure the trustworthiness of our qualitative content analysis, we focused on credibility, dependability, conformability and transferability. The credibility of the study was enhanced by the implementation of a systematic and transparent data collection process, which ensured the inclusion of diverse and representative patient narratives. To ensure the reliability and validity of the findings, we employed investigator triangulation, whereby multiple researchers independently coded and analysed the data. This approach was adopted to guarantee consistency and resolve any discrepancies that may have arisen through collaborative discussion. The analysis was grounded in detailed participant descriptions. The dependability of the research was ensured through meticulous documentation and consistency checks, which maintained a clear audit trail from data collection to analysis. Regular team reviews contributed to maintaining coding consistency, thereby reducing variability. Transferability was facilitated by the provision of rich contextual descriptions, thereby enabling readers to assess the applicability of the findings to other settings. The conformability of the analytical procedures was demonstrated by the clear delineation of said procedures, thus enabling the readers to evaluate the objectivity of the conclusions.

### 2.6 Ethical Statement

The study was reviewed and approved by the Ethics Committee of Qom University of Medical Sciences. Participation in the study was entirely voluntary, with all participants providing informed consent before participation. No financial or material incentives were provided. The study adhered to ethical guidelines for research involving human subjects, with confidentiality maintained and all data anonymised before analysis.

## 3 Results

The analysis is organised around three primary themes: Experiences of Coercion, Roots of Coercion and Effects of Coercive Measures. Each theme is further broken down into relevant sub‐themes to provide a comprehensive understanding of the participants' experiences. Illustrative quotes supporting each sub‐theme are presented in Table 3 for clarity and ease of reading.

**Table 3:** Themes, sub‐themes and illustrative quotes from participants' experiences of coercion.

Themes	Sub‐themes	Representative quotes
Experiences of Coercion	Coercive practices in psychiatric wards	…“I asked them to give me an injection instead of restraining me. I lay on my stomach. They talked to each other, and when they agreed, I was a little relieved because I hated being tied up” (P 1). …“They wanted to shave my beard. I didn't want to, but they said I couldn't keep myself clean if I didn't. In the end, they pressured me so much that I had to let them shave my beard” (P 5). …“The nurse wouldn't let me watch TV for more than an hour, even if I wanted to. She said, ‘If you want to stay up, you have to stay in your bed” (P 12).
	Coercive atmosphere	…“Everything here is mandatory, and it feels like a prison. At least when you're in prison, you know you're a prisoner. Here, you come for treatment, and you see that some restrictions don't want to change. Even if you protest, you might be restrained” (P 4). …“I have no other choice. Everything here is required. You just have to do whatever they say” (P 7).
	Perceived preventability of coercion	…“If someone had talked to me about my concerns, maybe I would have calmed down with a phone call to my family and there would have been no need for injections or restraints” (P 6). …“Sometimes, you can avoid extreme measures like restraint by thinking about the small things that cause the patient to get angry” (P 10).
The Roots of Coercion	Poor therapeutic communication	…“When you talk to the doctor, he doesn't pay much attention. He just asks his usual questions and quickly moves on to the next patient. Then, you're left with a lot of unanswered questions and problems. If we could have talked better, I wouldn't have felt so trapped” (P 2). …“Once I asked a question and the nurse said, Go away, I don't have time. When I protested, she got even angrier and said, If you don't go, we'll restrain you” (P 11).
	Patient characteristics	…“During the first few days of my stay in the hospital, I didn't accept my illness. I was sceptical of some of my family members. I wanted to leave the hospital. I was restless, so I was tied up several times and given injections” (P 7). …“One time, when I was in a psychiatric ward, I saw a patient who had been forcibly admitted. He was very aggressive and threw the bedside cabinet, which scared all the other patients and disrupted the peace. The staff had to restrain him” (P 13).
	Staff behaviour	…“The nurses on this ward must be different from those on other wards. They must be able to treat psychiatric patients properly” (12). …“One of the patients said something to me that upset me and I told him to watch his behaviour and I grabbed his collar. when the nurses saw that, they quickly came and restrained me without investigating the cause. They just tied me up and didn't pay any attention to the root of the problem” (P 8).
	Environmental factors	…“There are limited hours for watching TV, and there are ridiculous rules about smoking and sleeping that you can't even choose” (P 1).
Consequences of Coercive Measures	Psychological and physical impact	…“When I see the nurse who tied me up or when I pass by the restraint room, I remember all those horrible scenes and feel sick. Sometimes I even have bad dreams about what happened” (P 5). …“While they were binding me, I was thinking that I wish I had killed myself. I would like to die but not be bound like that again” (P 7). …“When I was restrained. One of the guards pressed my leg so hard that it was bruised and painful for several days” (P 9).
	Patients' perceptions of psychiatric care	“The doctor even prescribed shock therapy for me without explaining it well enough. I don't think these treatments are useful” (P 13). “I told them that these medications don't work well for me and make me feel bad, but they keep telling me to take them. Even now, if it were up to me, I wouldn't take all of them because I don't think many of them are useful” (P 10).
	Violation of dignity	…“When they tried to tie me up, they treated me very badly. I'm not an animal. They tied me up like a monkey” (P 5). …“When I was tied up, I said, Untie me. I have to go to the bathroom. If I don't, I'm going to wet myself. One of the nurses said, We're not going to untie you. You're going to have to pee on yourself” (P 6).

### 3.1 Experiences of Coercion

This theme examines patients' experiences of coercion in psychiatric wards, encompassing both formal and informal measures. It highlights the emotional impact of these practices, the coercive atmosphere of the ward and patients' perceptions of how such measures could have been prevented. These experiences are represented through three sub‐themes: types of coercive measures, coercive atmosphere and perceived preventability of coercion.

### 3.2 Coercive Practices in Psychiatric Wards

This sub‐theme reflects patients' experiences of both formal and informal coercive measures. Formal measures included involuntary hospitalisation, forced medication, mechanical restraints and physical restraints, with seclusion reported rarely. Chemical restraint was generally perceived as more acceptable than mechanical restraint, as indicated by patients' emotional responses. Informal measures involved enforced haircuts (primarily for hygiene, although some patients without hygiene issues were also subjected to this measure), rigorous unwritten rules (e.g., limited phone access, restricted recreational activities, mandatory chores and limited TV time) and harsh verbal encouragement (e.g., shouting, scolding or pressuring patients to follow rules). These practices represent environmental and organisational constraints shaping daily ward life and often constitute hidden or structural coercion, implemented unconsciously by staff, such as restricting access to activities or privileges.

### 3.3 Coercive Atmosphere

The coercive nature of many of the measures used in psychiatric wards often resulted in deep feelings of entrapment, helplessness and neglect on the part of ward staff, and many patients saw hospitalisation as a kind of incarceration, with rigid rules and routines, poor explanations of treatment procedures, lack of patient consent and lack of personal choice, often linked to poor engagement with healthcare providers.

### 3.4 Perceived Preventability of Coercion

Most patients perceived that, in many cases, coercive measures could have been avoided or reduced. The preventive strategies perceived by patients included establishing a strong therapeutic relationship, addressing the underlying causes of patients' irritability, providing clear explanations regarding the ward environment and treatment procedures, and implementing flexible routines and recreational activities within the ward.

### 3.5 The Roots of Coercion

This theme addresses the multifaceted roots of coercion, encompassing ineffective communication, patient‐related factors, staff behaviours and environmental conditions. These four interrelated sub‐themes illustrate the complex decision‐making dynamics that culminate in the use of coercive measures.

### 3.6 Poor Therapeutic Communication

Patients frequently reported that the quality of therapeutic communication with staff was suboptimal. They emphasised that improving communication could help reduce the use of coercive measures and the experience of coercion. Many patients reported feeling that their opinions were undervalued and that staff made insufficient efforts to engage them in meaningful dialogue.

### 3.7 Patient Characteristics

The majority of patients indicated that aggression and harm to themselves and others constituted a justification for the utilisation of coercive measures, such as restraint and forced medication. The lack of insight regarding the disease and the nature of the psychiatric disorder was identified as a contributing factor to irritability and aggression.

### 3.8 Staff Behaviour

In general, inappropriate behaviour of nurses and other ward staff contributed to patient irritability and increased the likelihood of coercive measures. Patients reported that staff sometimes acted hastily, implementing physical or chemical restraints before fully assessing the situation or attempting alternative interventions. Other problematic behaviours included humiliating behaviour (e.g., mocking or belittling patients) and discriminatory behaviour (e.g., applying ward rules unequally for different patients), as well as responding aggressively to patient inquiries and insufficient attention to patients' concerns. These actions were perceived to escalate conflicts rather than resolve underlying issues, highlighting the need for careful and respectful engagement by all ward personnel.

### 3.9 Environmental Factors

The physical and social environment of psychiatric wards significantly influenced patients' experiences of coercion. Limited physical space meant that a single hall was shared for multiple purposes, including smoking, dining and watching TV, which sometimes caused discomfort and tension among patients. Recreational facilities to occupy patients' leisure time were also insufficient; resources such as books, TV, music and basic indoor exercise equipment were limited. Patients often experienced the ward as crowded and congested. Mixing patients with different symptom severity levels further heightened irritability and aggressive behaviours. Strict phone rules and limited family visit spaces also contributed to feelings of confinement. Overall, patients perceived that these environmental limitations increased their distress and sometimes led to situations where coercive measures were used.

## 4 Consequences of Coercive Measures

This theme captures the outcomes of coercive measures on patients, including negative psychological effects, altered attitudes towards psychiatric care and experiences of violated dignity. These lasting emotional and behavioural consequences are organised into three sub‐themes.

### 4.1 Psychological and Physical Impact of Coercion

Participants described coercive measures as having negative effects, often causing psychological harm like anger, worthlessness, fear and anxiety. Physical and mechanical restraints were seen as more severe, sometimes leading to suicidal thoughts. Many recalled restraints as deeply unpleasant, and physical injuries or discomfort during restraint were also reported as adverse outcomes.

### 4.2 Patients' Perceptions of Psychiatric Care

Patients reported that coercive measures negatively shaped their perceptions of psychiatric care. Common experiences included reduced trust in staff, reluctance to follow treatments and avoidance of future care. These accounts highlight how coercion can undermine patients' engagement with and confidence in psychiatric care.

### 4.3 Violation of Dignity

Participants reported dissatisfaction with their treatment, indicating that they were not afforded the respect and dignity typically granted to individuals. They frequently encountered violations of their human dignity, which included a disregard for their autonomy, exclusion from treatment programmes and experiences related to severe coercive measures such as involuntary hospitalisation, physical restraint and forced medication.

**Figure 1:**
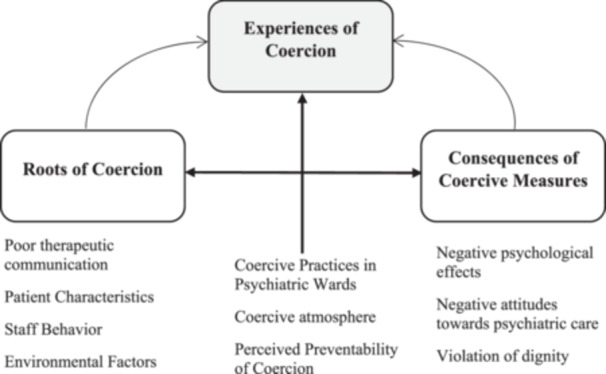
Conceptual framework showing interconnections between themes and sub‐themes of patients' experiences of coercion.

The relationships among themes and sub‐themes are summarised in Figure 1, illustrating how coercive measures in psychiatric wards, their underlying roots and resulting consequences interact to shape patients' experiences of coercion.

## 5 Discussion

Coercive measures are frequently employed in locked psychiatric wards to manage aggressive behaviour and ensure the safety of patients and staff. These interventions, which can encompass a range of formal and informal measures, raise ethical concerns regarding patient autonomy and the therapeutic milieu [15, 20]. This qualitative study examined the experiences of psychiatric patients subjected to coercive measures in locked wards, identifying three overarching themes: The following topics will be examined: experiences of coercion, roots of coercive measures and consequences of coercive measures.

The application of coercion in psychiatric care can be broadly categorised into formal and informal measures. Formal coercion refers to legally sanctioned and documented interventions, such as involuntary hospitalisation, forced medication, mechanical or physical restraint, and seclusion, typically applied in response to safety concerns. In contrast, informal coercion encompasses non‐legally mandated pressures, including verbal persuasion, threats, and hidden or structural practices, such as rigid unwritten rules, restricted access to activities or limitations on daily routines. These informal practices may be implemented consciously or unconsciously by staff and, while less visible than formal measures, can substantially affect patients' autonomy, dignity and experience of care [8]. Research findings indicate that mechanical restraints are the most prevalent coercive measures in locked psychiatric settings, frequently resulting in significant psychological distress for patients [2]. The majority of patients express a preference for medication over physical restraint, perceiving it as a less intrusive and more efficacious approach for managing symptoms while maintaining autonomy and dignity. Research indicates that this inclination is particularly pronounced among individuals who have encountered both methods [21, 22]. Participants frequently perceived psychiatric wards to be coercive and akin to prison‐like environments. While coercive measures are occasionally necessary for safety, their deleterious effects on patients' mental health and ward atmosphere underscore the necessity for alternative approaches [23, 24]. Research findings indicate that treatment in wards characterised by an open‐door policy is associated with a reduced frequency of coercive measures. Patients who were initially admitted to open‐door wards and subsequently transferred to closed‐door wards exhibited a higher probability of encountering coercion. This finding underscores the pivotal role of the ward environment in determining the necessity and frequency of coercive measures [15, 25].

The present study's findings suggest that patients believe coercive measures in psychiatric wards are preventable. Our results indicate that establishing appropriate therapeutic communication and addressing the underlying factors contributing to these measures can reduce their use. This aligns with existing research demonstrating that clear and empathetic communication can mitigate patient agitation and decrease the likelihood of coercion [26]. Reducing coercive measures can also be achieved through targeted interventions and the strategic implementation of evidence‐based guidelines. Staff training in de‐escalation techniques, structured risk assessments and the cultivation of a supportive ward environment through regular workshops and team meetings are all effective strategies to prevent crises that may lead to coercion [21, 27, 28, 29, 30].

Several innovative inpatient care models, including open‐door wards, patient‐centred approaches, structured de‐escalation programmes and interventions such as Safewards, the Six Core Strategies and REsTRAIN, have been shown to reduce coercive measures. Combining these with traditional preventive strategies can create a safer, less coercive environment, enhance patient engagement, and support staff in managing challenging situations [17, 27, 31]. The findings of this study indicate that coercive measures employed in psychiatric wards are influenced by a multifaceted interplay of factors associated with patients, staff and the ward environment. The study identified suboptimal therapeutic communication as a significant factor leading to coercive measures. This finding aligns with the conclusions of several studies that have highlighted the exacerbating effect of poor communication on patient distress, consequently leading to an escalation in the utilisation of restraints. Consequently, enhancing staff communication skills has been demonstrated to result in a reduction in the utilisation of restraints [32, 33]. Patients demonstrating aggressive behaviour before admission are more likely to undergo coercive measures. Research findings suggest that these patients face a threefold elevated risk compared to non‐aggressive individuals [34]. The severity of a patient's psychiatric condition plays a critical role. It has been demonstrated that each escalation in severity is associated with a twofold increase in the probability of coercion. This underscores the imperative for meticulous evaluation and surveillance of patients deemed to be at elevated risk [35]. The physical environment of psychiatric wards affects the use of coercive measures. Poor infrastructure, like a lack of private or outdoor spaces, increases patient anxiety and conflict, leading to more coercion. Additionally, restrictive institutional policies and emergency protocols can create situations requiring coercive interventions [36, 37].

The use of coercive measures in psychiatric wards poses significant risks to patient well‐being and treatment outcomes [38]. The psychological impact of coercive measures was profound, with many subjects reporting the experience of psychological distress following coercion, including feelings of anger, worthlessness, fear and anxiety. This finding aligns with extant literature that indicates that coercion can lead to long‐term psychological harm, including post‐traumatic stress symptoms [4, 39, 40]. A recent study revealed that psychiatric inpatients who had experienced coercion exhibited a significantly higher risk of suicide attempts following discharge compared to other patients who had not experienced coercion [41]. A systematic review also demonstrated that hospitalisation in psychiatric wards is associated with an elevated risk of post‐discharge suicide. This study underscores the long‐term repercussions of coercive interventions on suicide risk [42].

A sub‐theme of the effects of coercive measures theme was negative attitudes towards psychiatric care. The patients' attitudes towards psychiatric care can be negatively affected by their encounters with coercive measures. Most patients have reported feelings of trauma and distrust following such interventions. The psychological impact of coercive measures can be profound, often resulting in long‐lasting negative perceptions of psychiatric treatment, which may lead to a reluctance to seek help in the future [43, 44]. Moreover, research findings suggest that patients who experience coercion may interpret their treatment as punitive rather than therapeutic. This can intensify feelings of helplessness and hopelessness [4, 6, 45].

Coercive measures in psychiatric settings, while often justified as protective actions, can paradoxically lead to violations of patients' dignity and autonomy. Such interventions may contribute to feelings of humiliation, helplessness and reduced self‐esteem [33, 46, 47]. This cyclical pattern highlights that even safety‐focused measures, if implemented without attention to patients' rights and personal agency, can produce substantial psychological distress and undermine the therapeutic environment [46, 48, 49].

Incorporating evidence‐based frameworks such as Trauma‐Informed Care (TIC) and Recovery‐Oriented Practice (ROP) may offer effective strategies to reduce coercive measures in psychiatric wards. These approaches emphasise safety, empowerment and collaboration, aligning with the study's findings on the importance of empathetic communication, respect for dignity and individualised care [18].

## 6 Conclusion

The findings of this study emphasise the necessity for systemic changes within psychiatric wards to address the issues surrounding coercive measures. By prioritising effective communication, understanding individual patient needs, enhancing staff training and optimising the physical environment, psychiatric care can evolve towards a model that respects patient dignity while effectively managing challenging behaviours. The insights gained from this study should inform policy development aimed at minimising coercion and improving patient outcomes. These efforts should be grounded in evidence‐based frameworks such as TIC and ROP, which emphasise safety, empowerment and collaboration as fundamental principles of psychiatric treatment.

## Author Contributions

**Hamid Asayesh:** conceptualisation, methodology, formal analysis, investigation, validation, writing – review and editing. **Mohammad Parvaresh‐masoud:** investigation, data curation, methodology, formal analysis, writing – original draft, writing – review and editing. **Vahideh Nayeri:** resources, clinical support, validation, writing – review and editing. **Ali Khaji:** theoretical contribution, validation, methodology, writing – review and editing. **Ahmad Mashkoori:** conceptualisation, methodology, supervision, project administration, formal analysis, writing – original draft, writing – review and editing.

## Ethics Statement

The study was approved by the Ethics Committee of Qom University of Medical Sciences. Participation was voluntary, and no incentives were provided. Confidentiality was ensured, and all data were anonymised before analysis.

## Consent

Informed consent was obtained from all participants.

## Conflicts of Interest

The authors declare no conflicts of interest.

## Data Availability

The datasets generated and analysed during this qualitative study are not publicly available due to ethical restrictions and confidentiality agreements protecting participant privacy. Anonymised excerpts from the interviews are included in the manuscript to support the findings. Further inquiries about the data can be directed to the corresponding author upon reasonable request.
